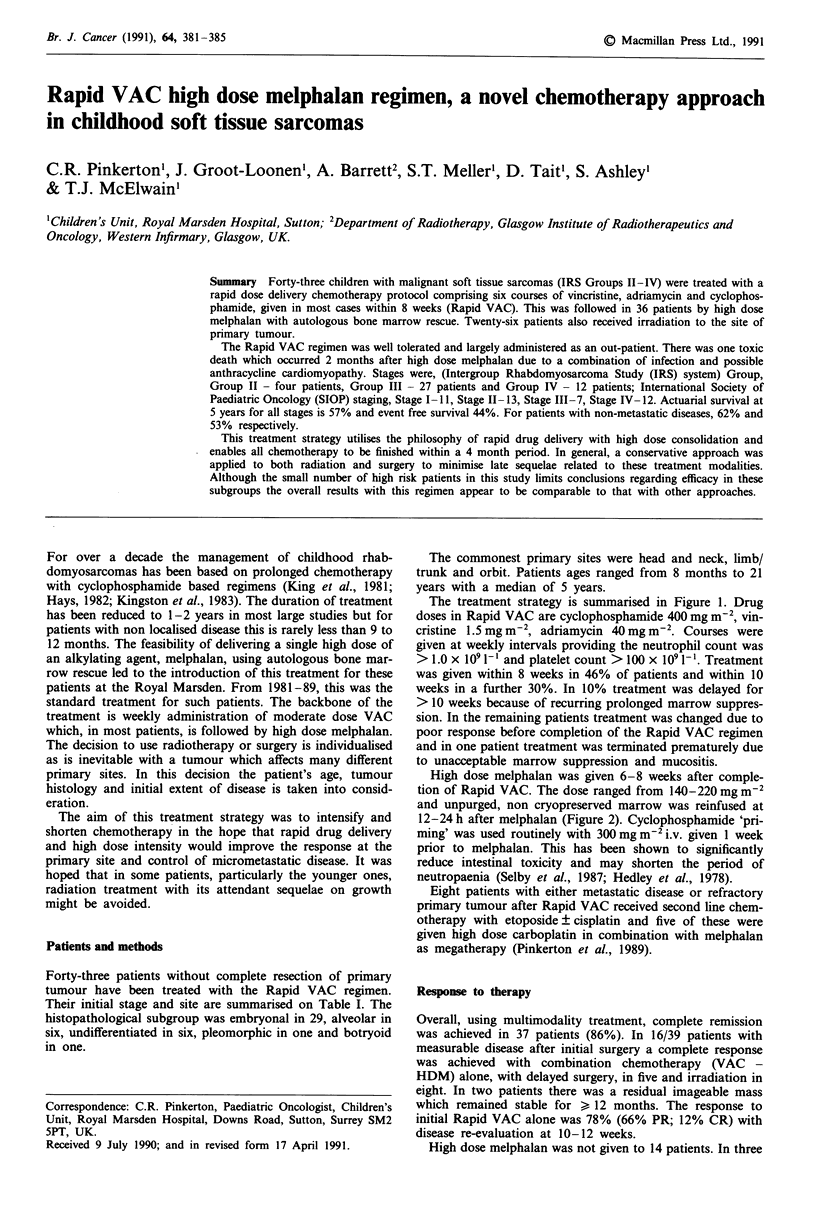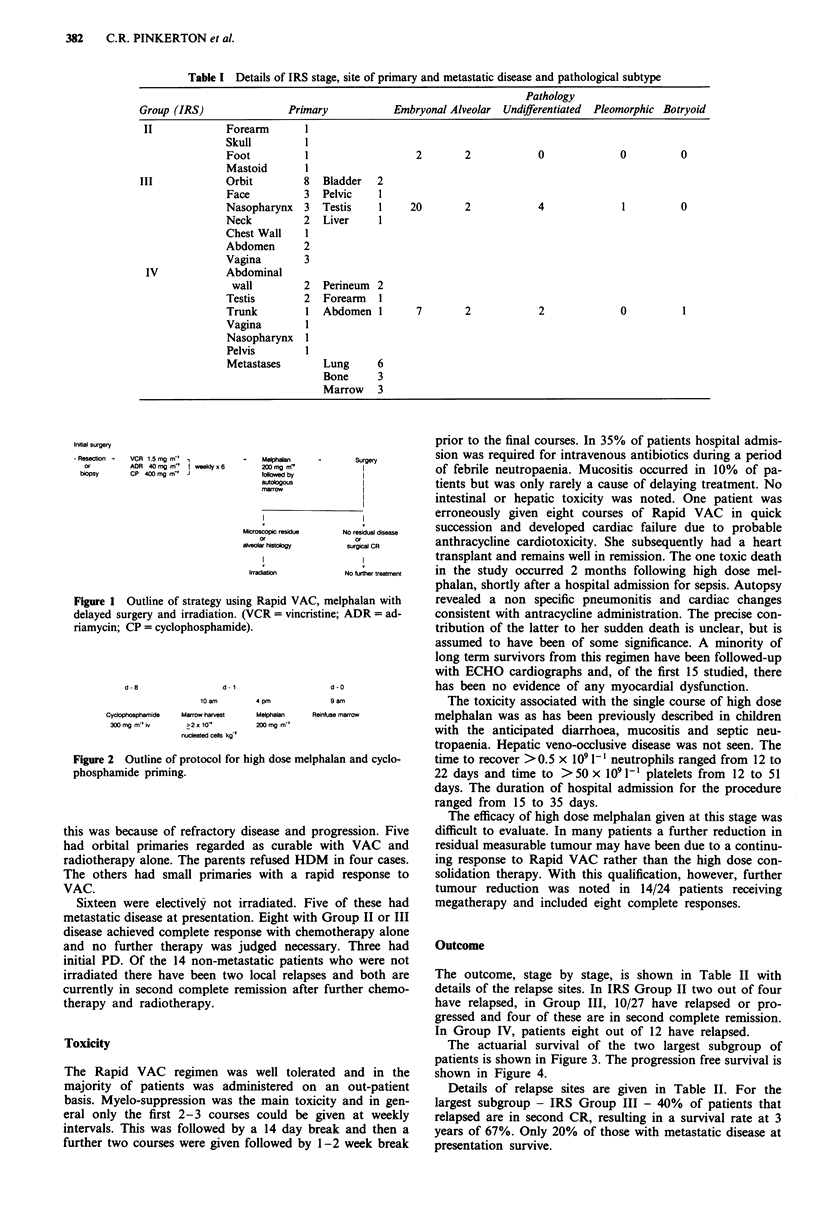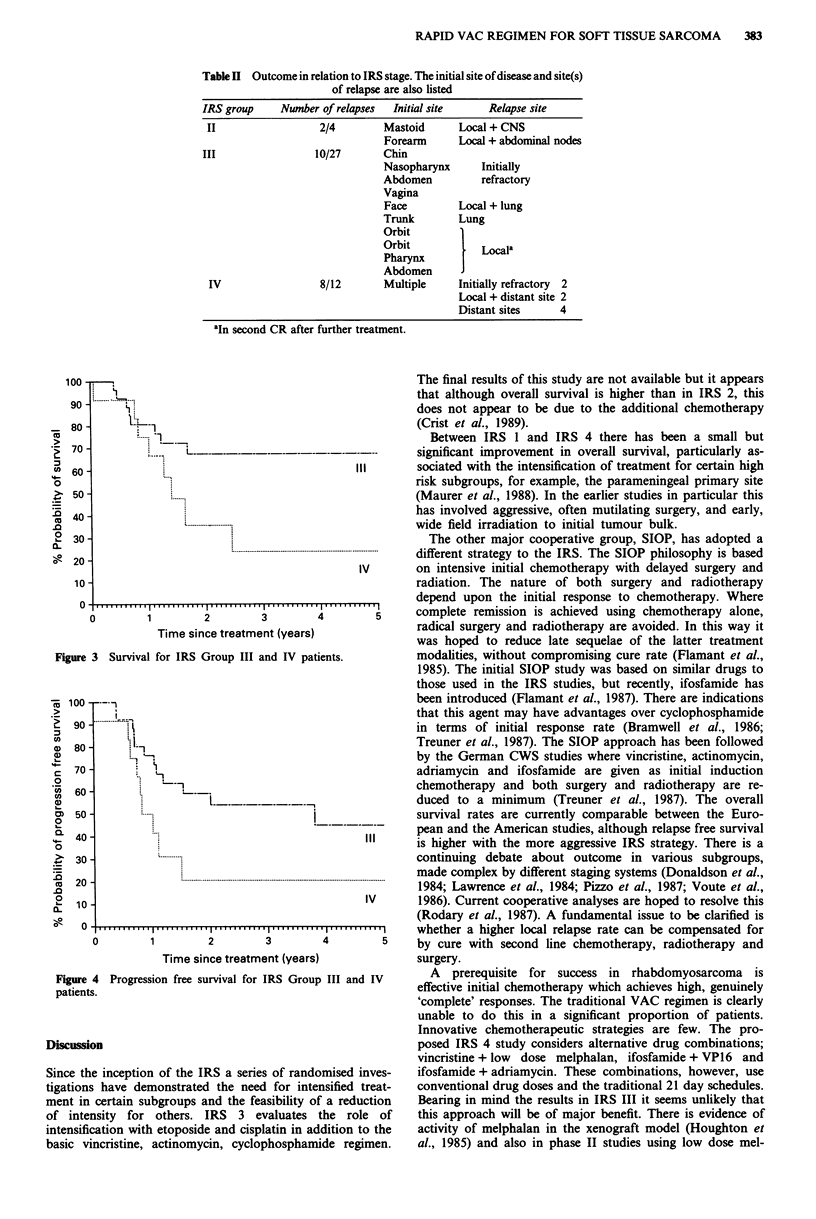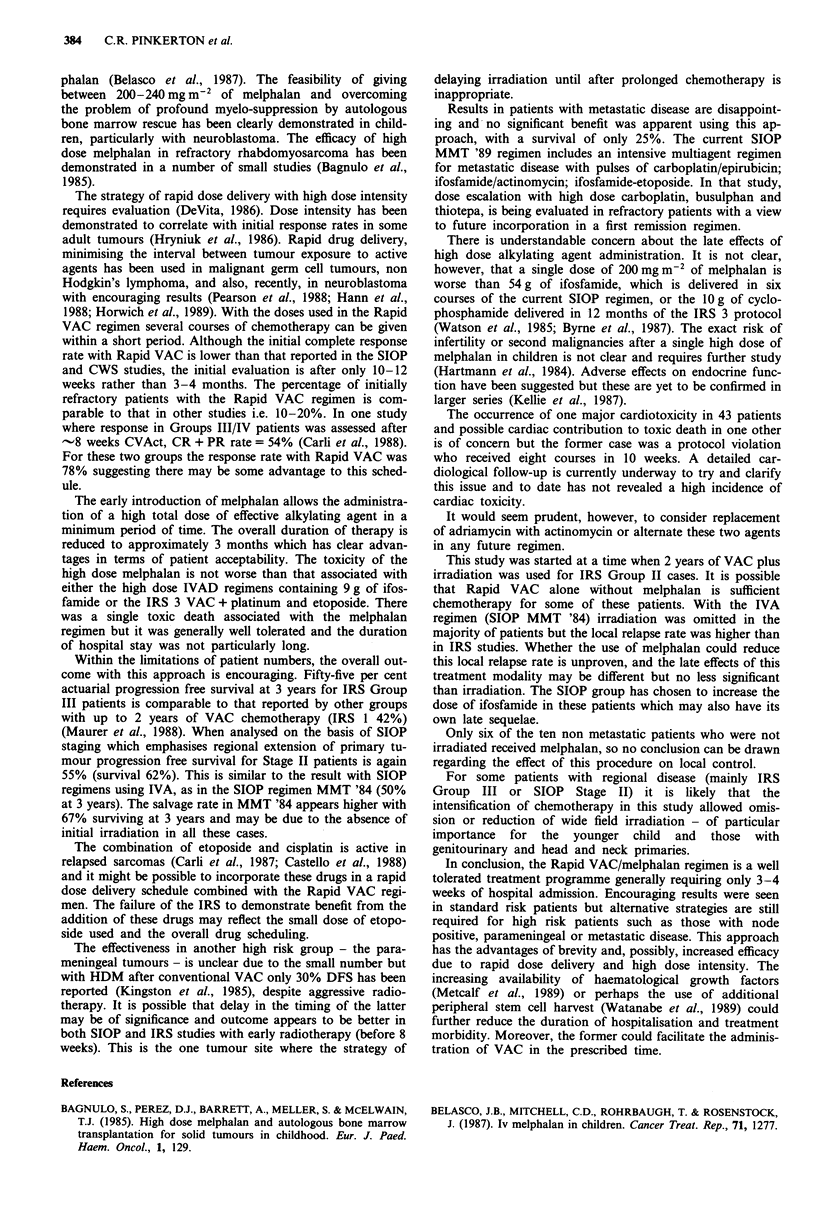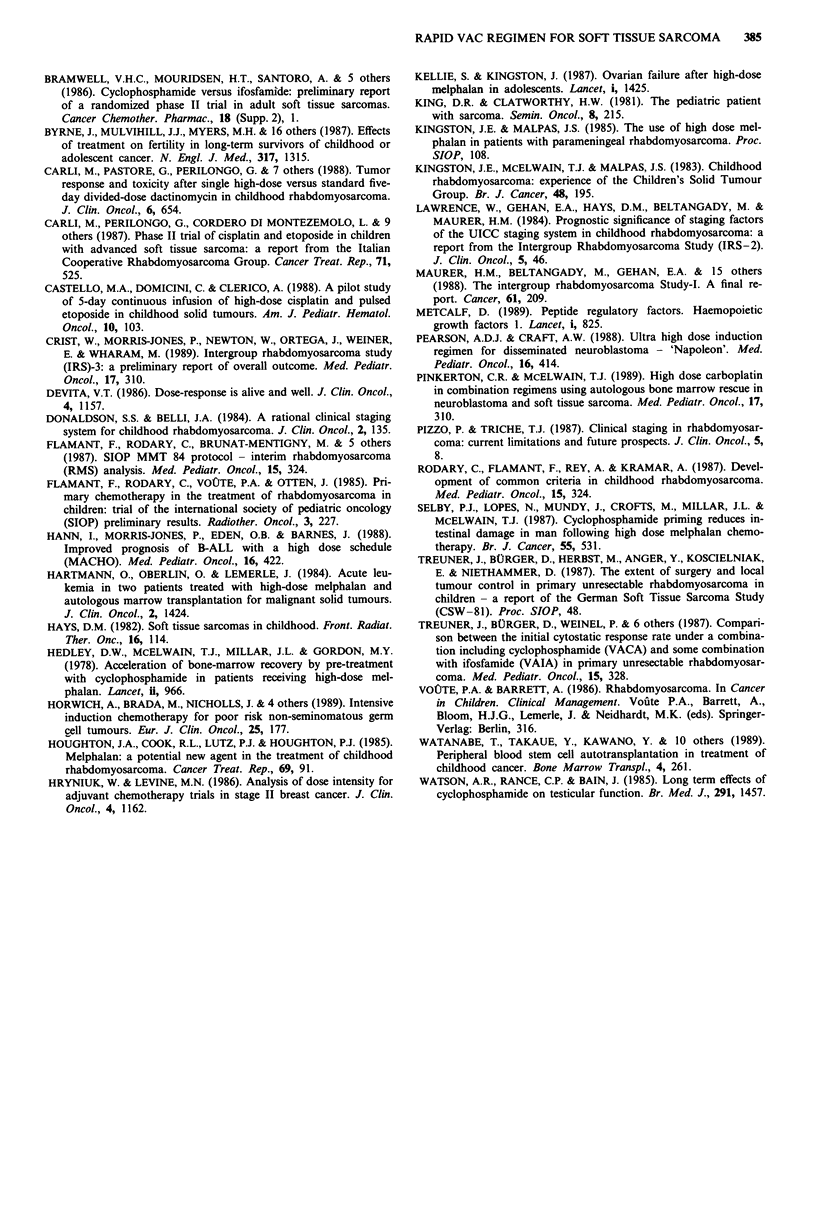# Rapid VAC high dose melphalan regimen, a novel chemotherapy approach in childhood soft tissue sarcomas.

**DOI:** 10.1038/bjc.1991.313

**Published:** 1991-08

**Authors:** C. R. Pinkerton, J. Groot-Loonen, A. Barrett, S. T. Meller, D. Tait, S. Ashley, T. J. McElwain

**Affiliations:** Children's Unit, Royal Marsden Hospital, Sutton, UK.

## Abstract

Forty-three children with malignant soft tissue sarcomas (IRS Groups II-IV) were treated with rapid dose delivery chemotherapy protocol comprising six courses of vincristine, adriamycin and cyclophosphamide, given in most cases within 8 weeks (Rapid VAC). This was followed in 36 patients by high dose melphalan with autologous bone marrow rescue. Twenty-six patients also received irradiation to the site of primary tumour. The Rapid VAC regimen was well tolerated and largely administered as an out-patient. There was one toxic death which occurred 2 months after high dose melphalan due to a combination of infection and possible anthracycline cardiomyopathy. Stages were, (Intergroup Rhabdomyosarcoma Study (IRS) system) Group, Group II--four patients. Group III--27 patients and Group IV--12 patients; International Society of Paediatric Oncology (SIOP) staging, Stage I--11, Stage II--13, Stage III--7, Stage IV--12. Actuarial survival at 5 years for all stages is 57% and event free survival 44%. For patients with non-metastatic diseases, 62% and 53% respectively. This treatment strategy utilises the philosophy of rapid drug delivery with high dose consolidation and enables all chemotherapy to be finished within a 4 month period. In general, a conservative approach was applied to both radiation and surgery to minimise late sequelae related to these treatment modalities. Although the small number of high risk patients in this study limits conclusions regarding efficacy in these subgroups the overall results with this regimen appear to be comparable to that with other approaches.


Br. J. Cancer (1991), 64, 381-385 © Macmillan Press Ltd., 1991

### Rapid VAC high dose melphalan regimen, a novel chemotherapy approach in childhood soft tissue sarcomas

C.R. Pinkerton[1], J. Groot-Loonen[1], A. Barrett[2], S.T. Meller[1], D. Tait[1], S. Ashley[1] & T.J. McElwain[1]

[1]Children's Unit, Royal Marsden Hospital, Sutton; [2]Department of Radiotherapy, Glasgow Institute of Radiotherapeutics and Oncology, Western Infirmary, Glasgow, UK.

**Summary** Forty-three children with malignant soft tissue sarcomas (IRS Groups II–IV) were treated with a rapid dose delivery chemotherapy protocol comprising six courses of vincristine, adriamycin and cyclophosphamide, given in most cases within 8 weeks (Rapid VAC). This was followed in 36 patients by high dose melphalan with autologous bone marrow rescue. Twenty-six patients also received irradiation to the site of primary tumour.

The Rapid VAC regimen was well tolerated and largely administered as an out-patient. There was one toxic death which occurred 2 months after high dose melphalan due to a combination of infection and possible anthracycline cardiomyopathy. Stages were, (Intergroup Rhabdomyosarcoma Study (IRS) system) Group, Group II – four patients, Group III – 27 patients and Group IV – 12 patients; International Society of Paediatric Oncology (SIOP) staging, Stage I–11, Stage II–13, Stage III–7, Stage IV–12. Actuarial survival at 5 years for all stages is 57% and event free survival 44%. For patients with non-metastatic diseases, 62% and 53% respectively.

This treatment strategy utilises the philosophy of rapid drug delivery with high dose consolidation and enables all chemotherapy to be finished within a 4 month period. In general, a conservative approach was applied to both radiation and surgery to minimise late sequelae related to these treatment modalities. Although the small number of high risk patients in this study limits conclusions regarding efficacy in these subgroups the overall results with this regimen appear to be comparable to that with other approaches.

For over a decade the management of childhood rhabdomyosarcomas has been based on prolonged chemotherapy with cyclophosphamide based regimens (King *et al.*, 1981; Hays, 1982; Kingston *et al.*, 1983). The duration of treatment has been reduced to 1–2 years in most large studies but for patients with non localised disease this is rarely less than 9 to 12 months. The feasibility of delivering a single high dose of an alkylating agent, melphalan, using autologous bone marrow rescue led to the introduction of this treatment for these patients at the Royal Marsden. From 1981–89, this was the standard treatment for such patients. The backbone of the treatment is weekly administration of moderate dose VAC which, in most patients, is followed by high dose melphalan. The decision to use radiotherapy or surgery is individualised as is inevitable with a tumour which affects many different primary sites. In this decision the patient's age, tumour histology and initial extent of disease is taken into consideration.

The aim of this treatment strategy was to intensify and shorten chemotherapy in the hope that rapid drug delivery and high dose intensity would improve the response at the primary site and control of micrometastatic disease. It was hoped that in some patients, particularly the younger ones, radiation treatment with its attendant sequelae on growth might be avoided.

### Patients and methods

Forty-three patients without complete resection of primary tumour have been treated with the Rapid VAC regimen. Their initial stage and site are summarised on Table I. The histopathological subgroup was embryonal in 29, alveolar in six, undifferentiated in six, pleomorphic in one and botryoid in one.

Correspondence: C.R. Pinkerton, Paediatric Oncologist, Children's Unit, Royal Marsden Hospital, Downs Road, Sutton, Surrey SM2 5PT, UK.
Received 9 July 1990; and in revised form 17 April 1991.

The commonest primary sites were head and neck, limb/trunk and orbit. Patients ages ranged from 8 months to 21 years with a median of 5 years.

The treatment strategy is summarised in Figure 1. Drug doses in Rapid VAC are cyclophosphamide 400 mg $m^{-2}$, vincristine 1.5 mg $m^{-2}$, adriamycin 40 mg $m^{-2}$. Courses were given at weekly intervals providing the neutrophil count was $> 1.0 \times 10^9 l^{-1}$ and platelet count $> 100 \times 10^9 l^{-1}$. Treatment was given within 8 weeks in 46% of patients and within 10 weeks in a further 30%. In 10% treatment was delayed for $> 10$ weeks because of recurring prolonged marrow suppression. In the remaining patients treatment was changed due to poor response before completion of the Rapid VAC regimen and in one patient treatment was terminated prematurely due to unacceptable marrow suppression and mucositis.

High dose melphalan was given 6–8 weeks after completion of Rapid VAC. The dose ranged from 140–220 mg $m^{-2}$ and unpurged, non cryopreserved marrow was reinfused at 12–24 h after melphalan (Figure 2). Cyclophosphamide 'priming' was used routinely with 300 mg $m^{-2}$ i.v. given 1 week prior to melphalan. This has been shown to significantly reduce intestinal toxicity and may shorten the period of neutropaenia (Selby *et al.*, 1987; Hedley *et al.*, 1978).

Eight patients with either metastatic disease or refractory primary tumour after Rapid VAC received second line chemotherapy with etoposide ± cisplatin and five of these were given high dose carboplatin in combination with melphalan as megatherapy (Pinkerton *et al.*, 1989).

### Response to therapy

Overall, using multimodality treatment, complete remission was achieved in 37 patients (86%). In 16/39 patients with measurable disease after initial surgery a complete response was achieved with combination chemotherapy (VAC – HDM) alone, with delayed surgery, in five and irradiation in eight. In two patients there was a residual imageable mass which remained stable for $\geqslant 12$ months. The response to initial Rapid VAC alone was 78% (66% PR; 12% CR) with disease re-evaluation at 10–12 weeks.

High dose melphalan was not given to 14 patients. In three

**Table I** Details of IRS stage, site of primary and metastatic disease and pathological subtype

| Group (IRS) | Primary | | | | Pathology Embryonal | Alveolar | Undifferentiated | Pleomorphic | Botryoid |
|---|---|---|---|---|---|---|---|---|---|
| II | Forearm | 1 | | | | | | | |
| | Skull | 1 | | | | | | | |
| | Foot | 1 | | | 2 | 2 | 0 | 0 | 0 |
| | Mastoid | 1 | | | | | | | |
| III | Orbit | 8 | Bladder | 2 | | | | | |
| | Face | 3 | Pelvic | 1 | | | | | |
| | Nasopharynx | 3 | Testis | 1 | 20 | 2 | 4 | 1 | 0 |
| | Neck | 2 | Liver | 1 | | | | | |
| | Chest Wall | 1 | | | | | | | |
| | Abdomen | 2 | | | | | | | |
| | Vagina | 3 | | | | | | | |
| IV | Abdominal wall | 2 | Perineum | 2 | | | | | |
| | Testis | 2 | Forearm | 1 | | | | | |
| | Trunk | 1 | Abdomen | 1 | 7 | 2 | 2 | 0 | 1 |
| | Vagina | 1 | | | | | | | |
| | Nasopharynx | 1 | | | | | | | |
| | Pelvis | 1 | | | | | | | |
| | Metastases | | Lung | 6 | | | | | |
| | | | Bone | 3 | | | | | |
| | | | Marrow | 3 | | | | | |

**Figure 1** Outline of strategy using Rapid VAC, melphalan with delayed surgery and irradiation. (VCR = vincristine; ADR = adriamycin; CP = cyclophosphamide).

**Figure 2** Outline of protocol for high dose melphalan and cyclophosphamide priming.

this was because of refractory disease and progression. Five had orbital primaries regarded as curable with VAC and radiotherapy alone. The parents refused HDM in four cases. The others had small primaries with a rapid response to VAC.

Sixteen were electively not irradiated. Five of these had metastatic disease at presentation. Eight with Group II or III disease achieved complete response with chemotherapy alone and no further therapy was judged necessary. Three had initial PD. Of the 14 non-metastatic patients who were not irradiated there have been two local relapses and both are currently in second complete remission after further chemotherapy and radiotherapy.

### Toxicity

The Rapid VAC regimen was well tolerated and in the majority of patients was administered on an out-patient basis. Myelo-suppression was the main toxicity and in general only the first 2–3 courses could be given at weekly intervals. This was followed by a 14 day break and then a further two courses were given followed by 1–2 week break prior to the final courses. In 35% of patients hospital admission was required for intravenous antibiotics during a period of febrile neutropaenia. Mucositis occurred in 10% of patients but was only rarely a cause of delaying treatment. No intestinal or hepatic toxicity was noted. One patient was erroneously given eight courses of Rapid VAC in quick succession and developed cardiac failure due to probable anthracycline cardiotoxicity. She subsequently had a heart transplant and remains well in remission. The one toxic death in the study occurred 2 months following high dose melphalan, shortly after a hospital admission for sepsis. Autopsy revealed a non specific pneumonitis and cardiac changes consistent with antracycline administration. The precise contribution of the latter to her sudden death is unclear, but is assumed to have been of some significance. A minority of long term survivors from this regimen have been followed-up with ECHO cardiographs and, of the first 15 studied, there has been no evidence of any myocardial dysfunction.

The toxicity associated with the single course of high dose melphalan was as has been previously described in children with the anticipated diarrhoea, mucositis and septic neutropaenia. Hepatic veno-occlusive disease was not seen. The time to recover $>0.5 \times 10^9 l^{-1}$ neutrophils ranged from 12 to 22 days and time to $>50 \times 10^9 l^{-1}$ platelets from 12 to 51 days. The duration of hospital admission for the procedure ranged from 15 to 35 days.

The efficacy of high dose melphalan given at this stage was difficult to evaluate. In many patients a further reduction in residual measurable tumour may have been due to a continuing response to Rapid VAC rather than the high dose consolidation therapy. With this qualification, however, further tumour reduction was noted in 14/24 patients receiving megatherapy and included eight complete responses.

### Outcome

The outcome, stage by stage, is shown in Table II with details of the relapse sites. In IRS Group II two out of four have relapsed, in Group III, 10/27 have relapsed or progressed and four of these are in second complete remission. In Group IV, patients eight out of 12 have relapsed.

The actuarial survival of the two largest subgroup of patients is shown in Figure 3. The progression free survival is shown in Figure 4.

Details of relapse sites are given in Table II. For the largest subgroup – IRS Group III – 40% of patients that relapsed are in second CR, resulting in a survival rate at 3 years of 67%. Only 20% of those with metastatic disease at presentation survive.

**Table II** Outcome in relation to IRS stage. The initial site of disease and site(s) of relapse are also listed

| *IRS group* | *Number of relapses* | *Initial site* | *Relapse site* |
|---|---|---|---|
| II | 2/4 | Mastoid | Local + CNS |
| | | Forearm | Local + abdominal nodes |
| III | 10/27 | Chin | |
| | | Nasopharynx | Initially refractory |
| | | Abdomen | |
| | | Vagina | |
| | | Face | Local + lung |
| | | Trunk | Lung |
| | | Orbit | Local[a] |
| | | Orbit | |
| | | Pharynx | |
| | | Abdomen | |
| IV | 8/12 | Multiple | Initially refractory 2 |
| | | | Local + distant site 2 |
| | | | Distant sites 4 |

[a]In second CR after further treatment.

**Figure 3** Survival for IRS Group III and IV patients.

**Figure 4** Progression free survival for IRS Group III and IV patients.

### Discussion

Since the inception of the IRS a series of randomised investigations have demonstrated the need for intensified treatment in certain subgroups and the feasibility of a reduction of intensity for others. IRS 3 evaluates the role of intensification with etoposide and cisplatin in addition to the basic vincristine, actinomycin, cyclophosphamide regimen. The final results of this study are not available but it appears that although overall survival is higher than in IRS 2, this does not appear to be due to the additional chemotherapy (Crist *et al.*, 1989).

Between IRS 1 and IRS 4 there has been a small but significant improvement in overall survival, particularly associated with the intensification of treatment for certain high risk subgroups, for example, the parameningeal primary site (Maurer *et al.*, 1988). In the earlier studies in particular this has involved aggressive, often mutilating surgery, and early, wide field irradiation to initial tumour bulk.

The other major cooperative group, SIOP, has adopted a different strategy to the IRS. The SIOP philosophy is based on intensive initial chemotherapy with delayed surgery and radiation. The nature of both surgery and radiotherapy depend upon the initial response to chemotherapy. Where complete remission is achieved using chemotherapy alone, radical surgery and radiotherapy are avoided. In this way it was hoped to reduce late sequelae of the latter treatment modalities, without compromising cure rate (Flamant *et al.*, 1985). The initial SIOP study was based on similar drugs to those used in the IRS studies, but recently, ifosfamide has been introduced (Flamant *et al.*, 1987). There are indications that this agent may have advantages over cyclophosphamide in terms of initial response rate (Bramwell *et al.*, 1986; Treuner *et al.*, 1987). The SIOP approach has been followed by the German CWS studies where vincristine, actinomycin, adriamycin and ifosfamide are given as initial induction chemotherapy and both surgery and radiotherapy are reduced to a minimum (Treuner *et al.*, 1987). The overall survival rates are currently comparable between the European and the American studies, although relapse free survival is higher with the more aggressive IRS strategy. There is a continuing debate about outcome in various subgroups, made complex by different staging systems (Donaldson *et al.*, 1984; Lawrence *et al.*, 1984; Pizzo *et al.*, 1987; Voute *et al.*, 1986). Current cooperative analyses are hoped to resolve this (Rodary *et al.*, 1987). A fundamental issue to be clarified is whether a higher local relapse rate can be compensated for by cure with second line chemotherapy, radiotherapy and surgery.

A prerequisite for success in rhabdomyosarcoma is effective initial chemotherapy which achieves high, genuinely 'complete' responses. The traditional VAC regimen is clearly unable to do this in a significant proportion of patients. Innovative chemotherapeutic strategies are few. The proposed IRS 4 study considers alternative drug combinations; vincristine + low dose melphalan, ifosfamide + VP16 and ifosfamide + adriamycin. These combinations, however, use conventional drug doses and the traditional 21 day schedules. Bearing in mind the results in IRS III it seems unlikely that this approach will be of major benefit. There is evidence of activity of melphalan in the xenograft model (Houghton *et al.*, 1985) and also in phase II studies using low dose melphalan (Belasco et al., 1987). The feasibility of giving between 200–240 mg m$^{-2}$ of melphalan and overcoming the problem of profound myelo-suppression by autologous bone marrow rescue has been clearly demonstrated in children, particularly with neuroblastoma. The efficacy of high dose melphalan in refractory rhabdomyosarcoma has been demonstrated in a number of small studies (Bagnulo et al., 1985).

The strategy of rapid dose delivery with high dose intensity requires evaluation (DeVita, 1986). Dose intensity has been demonstrated to correlate with initial response rates in some adult tumours (Hryniuk et al., 1986). Rapid drug delivery, minimising the interval between tumour exposure to active agents has been used in malignant germ cell tumours, non Hodgkin's lymphoma, and also, recently, in neuroblastoma with encouraging results (Pearson et al., 1988; Hann et al., 1988; Horwich et al., 1989). With the doses used in the Rapid VAC regimen several courses of chemotherapy can be given within a short period. Although the initial complete response rate with Rapid VAC is lower than that reported in the SIOP and CWS studies, the initial evaluation is after only 10–12 weeks rather than 3–4 months. The percentage of initially refractory patients with the Rapid VAC regimen is comparable to that in other studies i.e. 10–20%. In one study where response in Groups III/IV patients was assessed after ~8 weeks CVAct, CR + PR rate = 54% (Carli et al., 1988). For these two groups the response rate with Rapid VAC was 78% suggesting there may be some advantage to this schedule.

The early introduction of melphalan allows the administration of a high total dose of effective alkylating agent in a minimum period of time. The overall duration of therapy is reduced to approximately 3 months which has clear advantages in terms of patient acceptability. The toxicity of the high dose melphalan is not worse than that associated with either the high dose IVAD regimens containing 9 g of ifosfamide or the IRS 3 VAC + platinum and etoposide. There was a single toxic death associated with the melphalan regimen but it was generally well tolerated and the duration of hospital stay was not particularly long.

Within the limitations of patient numbers, the overall outcome with this approach is encouraging. Fifty-five per cent actuarial progression free survival at 3 years for IRS Group III patients is comparable to that reported by other groups with up to 2 years of VAC chemotherapy (IRS 1 42%) (Maurer et al., 1988). When analysed on the basis of SIOP staging which emphasises regional extension of primary tumour progression free survival for Stage II patients is again 55% (survival 62%). This is similar to the result with SIOP regimens using IVA, as in the SIOP regimen MMT '84 (50% at 3 years). The salvage rate in MMT '84 appears higher with 67% surviving at 3 years and may be due to the absence of initial irradiation in all these cases.

The combination of etoposide and cisplatin is active in relapsed sarcomas (Carli et al., 1987; Castello et al., 1988) and it might be possible to incorporate these drugs in a rapid dose delivery schedule combined with the Rapid VAC regimen. The failure of the IRS to demonstrate benefit from the addition of these drugs may reflect the small dose of etoposide used and the overall drug scheduling.

The effectiveness in another high risk group – the parameningeal tumours – is unclear due to the small number but with HDM after conventional VAC only 30% DFS has been reported (Kingston et al., 1985), despite aggressive radiotherapy. It is possible that delay in the timing of the latter may be of significance and outcome appears to be better in both SIOP and IRS studies with early radiotherapy (before 8 weeks). This is the one tumour site where the strategy of delaying irradiation until after prolonged chemotherapy is inappropriate.

Results in patients with metastatic disease are disappointing and no significant benefit was apparent using this approach, with a survival of only 25%. The current SIOP MMT '89 regimen includes an intensive multiagent regimen for metastatic disease with pulses of carboplatin/epirubicin; ifosfamide/actinomycin; ifosfamide-etoposide. In that study, dose escalation with high dose carboplatin, busulphan and thiotepa, is being evaluated in refractory patients with a view to future incorporation in a first remission regimen.

There is understandable concern about the late effects of high dose alkylating agent administration. It is not clear, however, that a single dose of 200 mg m$^{-2}$ of melphalan is worse than 54 g of ifosfamide, which is delivered in six courses of the current SIOP regimen, or the 10 g of cyclophosphamide delivered in 12 months of the IRS 3 protocol (Watson et al., 1985; Byrne et al., 1987). The exact risk of infertility or second malignancies after a single high dose of melphalan in children is not clear and requires further study (Hartmann et al., 1984). Adverse effects on endocrine function have been suggested but these are yet to be confirmed in larger series (Kellie et al., 1987).

The occurrence of one major cardiotoxicity in 43 patients and possible cardiac contribution to toxic death in one other is of concern but the former case was a protocol violation who received eight courses in 10 weeks. A detailed cardiological follow-up is currently underway to try and clarify this issue and to date has not revealed a high incidence of cardiac toxicity.

It would seem prudent, however, to consider replacement of adriamycin with actinomycin or alternate these two agents in any future regimen.

This study was started at a time when 2 years of VAC plus irradiation was used for IRS Group II cases. It is possible that Rapid VAC alone without melphalan is sufficient chemotherapy for some of these patients. With the IVA regimen (SIOP MMT '84) irradiation was omitted in the majority of patients but the local relapse rate was higher than in IRS studies. Whether the use of melphalan could reduce this local relapse rate is unproven, and the late effects of this treatment modality may be different but no less significant than irradiation. The SIOP group has chosen to increase the dose of ifosfamide in these patients which may also have its own late sequelae.

Only six of the ten non metastatic patients who were not irradiated received melphalan, so no conclusion can be drawn regarding the effect of this procedure on local control.

For some patients with regional disease (mainly IRS Group III or SIOP Stage II) it is likely that the intensification of chemotherapy in this study allowed omission or reduction of wide field irradiation – of particular importance for the younger child and those with genitourinary and head and neck primaries.

In conclusion, the Rapid VAC/melphalan regimen is a well tolerated treatment programme generally requiring only 3–4 weeks of hospital admission. Encouraging results were seen in standard risk patients but alternative strategies are still required for high risk patients such as those with node positive, parameningeal or metastatic disease. This approach has the advantages of brevity and, possibly, increased efficacy due to rapid dose delivery and high dose intensity. The increasing availability of haematological growth factors (Metcalf et al., 1989) or perhaps the use of additional peripheral stem cell harvest (Watanabe et al., 1989) could further reduce the duration of hospitalisation and treatment morbidity. Moreover, the former could facilitate the administration of VAC in the prescribed time.

### References

BAGNULO, S., PEREZ, D.J., BARRETT, A., MELLER, S. & MCELWAIN, T.J. (1985). High dose melphalan and autologous bone marrow transplantation for solid tumours in childhood. *Eur. J. Paed. Haem. Oncol.*, **1**, 129.

BELASCO, J.B., MITCHELL, C.D., ROHRBAUGH, T. & ROSENSTOCK, J. (1987). Iv melphalan in children. *Cancer Treat. Rep.*, **71**, 1277.

BRAMWELL, V.H.C., MOURIDSEN, H.T., SANTORO, A. & 5 others (1986). Cyclophosphamide versus ifosfamide: preliminary report of a randomized phase II trial in adult soft tissue sarcomas. *Cancer Chemother. Pharmac.*, **18** (Supp. 2), 1.

BYRNE, J., MULVIHILL, J.J., MYERS, M.H. & 16 others (1987). Effects of treatment on fertility in long-term survivors of childhood or adolescent cancer. *N. Engl. J. Med.*, **317**, 1315.

CARLI, M., PASTORE, G., PERILONGO, G. & 7 others (1988). Tumor response and toxicity after single high-dose versus standard five-day divided-dose dactinomycin in childhood rhabdomyosarcoma. *J. Clin. Oncol.*, **6**, 654.

CARLI, M., PERILONGO, G., CORDERO DI MONTEZEMOLO, L. & 9 others (1987). Phase II trial of cisplatin and etoposide in children with advanced soft tissue sarcoma: a report from the Italian Cooperative Rhabdomyosarcoma Group. *Cancer Treat. Rep.*, **71**, 525.

CASTELLO, M.A., DOMICINI, C. & CLERICO, A. (1988). A pilot study of 5-day continuous infusion of high-dose cisplatin and pulsed etoposide in childhood solid tumours. *Am. J. Pediatr. Hematol. Oncol.*, **10**, 103.

CRIST, W., MORRIS-JONES, P., NEWTON, W., ORTEGA, J., WEINER, E. & WHARAM, M. (1989). Intergroup rhabdomyosarcoma study (IRS)-3: a preliminary report of overall outcome. *Med. Pediatr. Oncol.*, **17**, 310.

DEVITA, V.T. (1986). Dose-response is alive and well. *J. Clin. Oncol.*, **4**, 1157.

DONALDSON, S.S. & BELLI, J.A. (1984). A rational clinical staging system for childhood rhabdomyosarcoma. *J. Clin. Oncol.*, **2**, 135.

FLAMANT, F., RODARY, C., BRUNAT-MENTIGNY, M. & 5 others (1987). SIOP MMT 84 protocol – interim rhabdomyosarcoma (RMS) analysis. *Med. Pediatr. Oncol.*, **15**, 324.

FLAMANT, F., RODARY, C., VOÛTE, P.A. & OTTEN, J. (1985). Primary chemotherapy in the treatment of rhabdomyosarcoma in children: trial of the international society of pediatric oncology (SIOP) preliminary results. *Radiother. Oncol.*, **3**, 227.

HANN, I., MORRIS-JONES, P., EDEN, O.B. & BARNES, J. (1988). Improved prognosis of B-ALL with a high dose schedule (MACHO). *Med. Pediatr. Oncol.*, **16**, 422.

HARTMANN, O., OBERLIN, O. & LEMERLE, J. (1984). Acute leukemia in two patients treated with high-dose melphalan and autologous marrow transplantation for malignant solid tumours. *J. Clin. Oncol.*, **2**, 1424.

HAYS, D.M. (1982). Soft tissue sarcomas in childhood. *Front. Radiat. Ther. Onc.*, **16**, 114.

HEDLEY, D.W., MCELWAIN, T.J., MILLAR, J.L. & GORDON, M.Y. (1978). Acceleration of bone-marrow recovery by pre-treatment with cyclophosphamide in patients receiving high-dose melphalan. *Lancet*, **ii**, 966.

HORWICH, A., BRADA, M., NICHOLLS, J. & 4 others (1989). Intensive induction chemotherapy for poor risk non-seminomatous germ cell tumours. *Eur. J. Clin. Oncol.*, **25**, 177.

HOUGHTON, J.A., COOK, R.L., LUTZ, P.J. & HOUGHTON, P.J. (1985). Melphalan: a potential new agent in the treatment of childhood rhabdomyosarcoma. *Cancer Treat. Rep.*, **69**, 91.

HRYNIUK, W. & LEVINE, M.N. (1986). Analysis of dose intensity for adjuvant chemotherapy trials in stage II breast cancer. *J. Clin. Oncol.*, **4**, 1162.

KELLIE, S. & KINGSTON, J. (1987). Ovarian failure after high-dose melphalan in adolescents. *Lancet*, **i**, 1425.

KING, D.R. & CLATWORTHY, H.W. (1981). The pediatric patient with sarcoma. *Semin. Oncol.*, **8**, 215.

KINGSTON, J.E. & MALPAS, J.S. (1985). The use of high dose melphalan in patients with parameningeal rhabdomyosarcoma. *Proc. SIOP*, 108.

KINGSTON, J.E., MCELWAIN, T.J. & MALPAS, J.S. (1983). Childhood rhabdomyosarcoma: experience of the Children's Solid Tumour Group. *Br. J. Cancer*, **48**, 195.

LAWRENCE, W., GEHAN, E.A., HAYS, D.M., BELTANGADY, M. & MAURER, H.M. (1984). Prognostic significance of staging factors of the UICC staging system in childhood rhabdomyosarcoma: a report from the Intergroup Rhabdomyosarcoma Study (IRS–2). *J. Clin. Oncol.*, **5**, 46.

MAURER, H.M., BELTANGADY, M., GEHAN, E.A. & 15 others (1988). The intergroup rhabdomyosarcoma Study-I. A final report. *Cancer*, **61**, 209.

METCALF, D. (1989). Peptide regulatory factors. Haemopoietic growth factors 1. *Lancet*, **i**, 825.

PEARSON, A.D.J. & CRAFT, A.W. (1988). Ultra high dose induction regimen for disseminated neuroblastoma – 'Napoleon'. *Med. Pediatr. Oncol.*, **16**, 414.

PINKERTON, C.R. & MCELWAIN, T.J. (1989). High dose carboplatin in combination regimens using autologous bone marrow rescue in neuroblastoma and soft tissue sarcoma. *Med. Pediatr. Oncol.*, **17**, 310.

PIZZO, P. & TRICHE, T.J. (1987). Clinical staging in rhabdomyosarcoma: current limitations and future prospects. *J. Clin. Oncol.*, **5**, 8.

RODARY, C., FLAMANT, F., REY, A. & KRAMAR, A. (1987). Development of common criteria in childhood rhabdomyosarcoma. *Med. Pediatr. Oncol.*, **15**, 324.

SELBY, P.J., LOPES, N., MUNDY, J., CROFTS, M., MILLAR, J.L. & MCELWAIN, T.J. (1987). Cyclophosphamide priming reduces intestinal damage in man following high dose melphalan chemotherapy. *Br. J. Cancer*, **55**, 531.

TREUNER, J., BÜRGER, D., HERBST, M., ANGER, Y., KOSCIELNIAK, E. & NIETHAMMER, D. (1987). The extent of surgery and local tumour control in primary unresectable rhabdomyosarcoma in children – a report of the German Soft Tissue Sarcoma Study (CSW–81). *Proc. SIOP*, 48.

TREUNER, J., BÜRGER, D., WEINEL, P. & 6 others (1987). Comparison between the initial cytostatic response rate under a combination including cyclophosphamide (VACA) and some combination with ifosfamide (VAIA) in primary unresectable rhabdomyosarcoma. *Med. Pediatr. Oncol.*, **15**, 328.

VOÛTE, P.A. & BARRETT, A. (1986). Rhabdomyosarcoma. In *Cancer in Children. Clinical Management*. Voûte P.A., Barrett, A., Bloom, H.J.G., Lemerle, J. & Neidhardt, M.K. (eds). Springer-Verlag: Berlin, 316.

WATANABE, T., TAKAUE, Y., KAWANO, Y. & 10 others (1989). Peripheral blood stem cell autotransplantation in treatment of childhood cancer. *Bone Marrow Transpl.*, **4**, 261.

WATSON, A.R., RANCE, C.P. & BAIN, J. (1985). Long term effects of cyclophosphamide on testicular function. *Br. Med. J.*, **291**, 1457.